# 3β-Hy­droxy­lup-20(29)-en-28-yl 1*H*-imidazole-1-carboxyl­ate

**DOI:** 10.1107/S160053681002489X

**Published:** 2010-06-30

**Authors:** R. C. Santos, A. Matos Beja, J. A. R. Salvador, J. A. Paixão

**Affiliations:** a Laboratório de Química Farmacêutica, Faculdade de Farmácia, Universidade de Coimbra, Pólo das Ciências da Saúde, Azinhaga de Santa Comba, P-3000-548 Coimbra, Portugal; b CEMDRX, Departamento de Física, Faculdade de Ciências e Tecnologia, Universidade de Coimbra, P-3004-516 Coimbra, Portugal

## Abstract

The title triterpene, C_34_H_52_N_2_O_3_, is a C-28 carbamate derivative of betulin prepared in a one-step reaction from the commercially available 1,1′-carbonyl­diimidazole (CDI). All rings are fused *trans*. The X-ray study shows the retention of the configuration of C-28 with respect to the known chiral centres of the molecule. In the crystal, the mol­ecules are O—H⋯O hydrogen bonded *via* the hy­droxy group and the carbonyl group of the carbamate function into chains running along the *c* axis. A quantum-mechanical *ab initio* Roothaan Hartree–Fock calculation of the equilibrium geometry of the isolated mol­ecule gives values for bond-lengths and valency angles close to the experimental values. The calculations also reproduce the mol­ecular conformation well, with calculated puckering parameters that agree well with the observed values.

## Related literature

For the synthesis of the title compound, see: Santos *et al.* (2009 ▶). For the biological activity of betulin and betulinic acid, see: Dzubak *et al.* (2006 ▶); Tolstikova *et al.* (2006 ▶); Petronelli *et al.* (2009 ▶). For plant triterpenes as potential anti-cancer drugs, see: Kinghorn *et al.* (2004 ▶); Setzer & Setzer (2003 ▶). For products afforded by the reaction of CDI with alcohols and phenols, see: Tang *et al.* (2004 ▶); Totleben *et al.* (1997 ▶); Herbez & Fischer (2005 ▶); Moreira *et al.* (2008 ▶); Ramos Silva *et al.* (2007 ▶). For puckering and asymmetry parameters, see: Cremer & Pople (1975 ▶); Duax & Norton (1975 ▶). The quantum chemical calculations were performed with the computer program *GAMESS* (Schmidt *et al.*, 1993 ▶). 
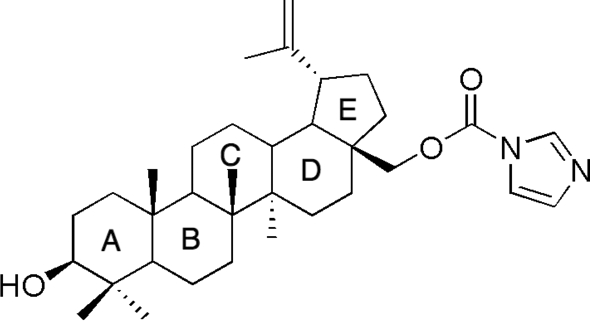


## Experimental

### 

#### Crystal data

C_34_H_52_N_2_O_3_
*M* *_r_* = 536.78
Orthorhombic, 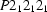

*a* = 8.2575 (2) Å
*b* = 12.3909 (4) Å
*c* = 29.0992 (8) Å
*V* = 2977.37 (15) Å^3^
*Z* = 4
Mo *K*α radiation
μ = 0.08 mm^−1^
*T* = 293 K
0.25 × 0.22 × 0.18 mm

#### Data collection

Bruker APEXII CCD area-detector diffractometer
Absorption correction: multi-scan (*SADABS*; Sheldrick, 2000 ▶) *T*_min_ = 0.898, *T*_max_ = 1.0
54547 measured reflections
3117 independent reflections
2106 reflections with *I* > 2σ(*I*)
*R*_int_ = 0.111

#### Refinement

*R*[*F* ^2^ > 2σ(*F* ^2^)] = 0.049
*wR*(*F* ^2^) = 0.119
*S* = 1.02
3117 reflections
360 parameters
H-atom parameters constrained
Δρ_max_ = 0.17 e Å^−3^
Δρ_min_ = −0.20 e Å^−3^

### 

Data collection: *APEX2*  (Bruker, 2006 ▶); cell refinement: *SAINT* (Bruker, 2006 ▶); data reduction: *SAINT*; program(s) used to solve structure: *SHELXS97* (Sheldrick, 2008 ▶); program(s) used to refine structure: *SHELXL97* (Sheldrick, 2008 ▶); molecular graphics: *PLATON* (Spek, 2009 ▶); software used to prepare material for publication: *SHELXL97*.

## Supplementary Material

Crystal structure: contains datablocks global, I. DOI: 10.1107/S160053681002489X/ez2217sup1.cif

Structure factors: contains datablocks I. DOI: 10.1107/S160053681002489X/ez2217Isup2.hkl

Additional supplementary materials:  crystallographic information; 3D view; checkCIF report

## Figures and Tables

**Table 1:** Hydrogen-bond geometry (Å, °)

*D*—H⋯*A*	*D*—H	H⋯*A*	*D*⋯*A*	*D*—H⋯*A*
O3*A*—H3*A*⋯O28*B*^i^	0.82	2.13	2.920 (4)	162

Symmetry code: (i) 
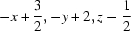
.

**Fig. 1.**
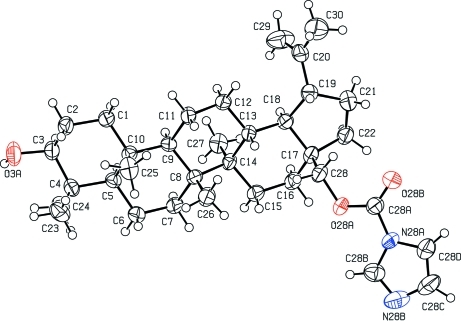
ORTEPII plot of the title compound showing the atomic numbering scheme. Displacement ellipsoids are drawn at the 50% level.

**Fig. 2.**
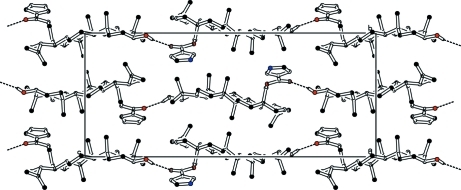
Packing diagram (view along the a axis) showing the hydrogen bonding network.

**Fig. 3.**
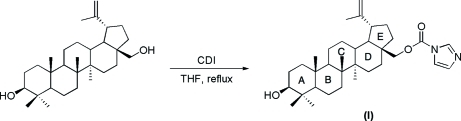
The formation of the title compound.

## supplementary crystallographic information

### Comment

Cancer is the second most important disease leading to death in both the developing and developed countries nowadays. Numerous experimental and epidemiological studies have shown that several plant derived natural products may serve as effective anticancer drugs, among which are plant triterpenes (Kinghorn *et al.*, 2004 and Setzer *et al.*, 2003). Betulin and betulinic acid, two pentacyclic lupane triterpenes were reported to display several biological effects including anti-inflammatory, antiviral, antimalarial and in particular anticancer (Dzubak *et al.*, 2006 and Tolstikova *et al.*, 2006). The therapeutic characteristics of betulinic acid regarding specificity and mode of action make it a promising anticancer agent presently under evaluation in phase I studies (Petronelli *et al.*, 2009).

As part of our current interest in the synthesis of new triterpenoid derivatives with cytotoxic activity, we have recently reported the synthesis and evaluation of novel carbamates and *N*-acylheterocyclic derivatives of betulin and betulinic acid for potential use as chemotherapeutic agents (Santos *et al.*, 2009).

The general procedure for the synthesis of the novel lupane derivatives involved dissolution of the corresponding lupanes and CDI, in THF at reflux, under N_2_. The reaction of CDI with alcohols and phenols has been reported to afford either *N*-alkylimidazoles (Tang *et al.*, 2004 and Totleben *et al.*, 1997) or imidazole carboxylic esters (carbamates) (Herbez *et al.*, 2005; Moreira *et al.*, 2008; Ramos Silva *et al.*, 2007; Tang *et al.*, 2004 and Totleben *et al.*, 1997) depending both on alcohol type and on the reaction conditions used. In this case the reaction afforded the carbamate derivative 3β-hydroxy-lup-20 (29)-en-28-yl-1*H*-imidazole-1-carboxylate in good yield. This compound had been found to induce a selective dose-dependent decrease in the viability of HepG2, HeLa and Jurkat cells after 72 h of treatment according to the determined IC_50_ values (4.2 µ M, 7.6 µ M and 16.3µ M, respectively), which were 2–8 times lower than that obtained with betulinic acid.

Mindful of the biological and synthetic importance of such molecules, we report in this communication the molecular structure of the 3β-hydroxy-lup-20 (29)-en-28-yl-1*H*-imidazole-1-carboxylate determined by single-crystal X-ray diffraction, and compare it with that of the free molecule as given by a quantum mechanical *ab initio* calculation. The structure of this compound with the corresponding atomic numbering scheme is shown in Fig. 1. This triterpenoid compound is a lupane-type with an imidazole carbonyloxy at C-28. The retention of configuration of C-28 was unequivocally demonstrated by this X-ray crystallographic study.

Bond lengths and valency angles have typical values for this type of compounds. All rings are fused *trans* as shown by the angle between the least-squares planes of the rings [rings A and B: 14.63 (18)°, B and C: 10.63 (18)°, C and D: 6.67 (18)°, D and E: 4.6 (2)°]. Rings A and C have conformations close to chair while rings B and D have conformations slightly distorted from chair towards half-chair as shown by the Cremer & Pople (1975) parameters [ring A: Q = 0.545 (4) Å, θ = 5.4 (4)° and φ = 36 (5)°; B: Q = 0.571 (4) Å, θ = 11.3 (4)° and φ = 1.0 (19)°; C: Q = 0.601 (4) Å, θ = 5.7 (4)° and φ = 338 (3)°; D: Q = 0.569 (4) Å, θ = 171.1 (4)° and φ = 90 (2)°]. Ring E has a twisted conformation along the C17–C18 bond [q_2_ = 0.443 (4) Å and φ_2_ = 9.0 (5)° and asymmetry parameters (Duax & Norton, 1975) ΔC_2_(C21) = ΔC_2_(C17,18) = 11.7 (4)°].

The molecules are hydrogen bonded involving the hydroxyl group at C3 and the carbonyl group of the carbamate moiety, forming infinite chains running along the *c* axis. In addition, two short distances between C16—H16A and C28—H28B and the O28A and O28B atoms, respectively may be due to weak intramolecular C—H···O interactions.

In order to gain some insight on how the crystal packing of (I) might affect the molecular geometry we have performed a quantum chemical calculation on the equilibrium geometry of the free molecule. These calculations were performed with the computer program GAMESS (Schmidt *et al.*, 1993).

The *ab initio* calculations reproduce the observed experimental bond lengths and valency angles of the molecule well, with the exception of the bond C20—C30 for which the calculations gave a distance of 1.5103 Å instead of the observed value of 1.433 (6) Å. Also, the calculated conformations of the rings are very close to the experimental values, with the exception of ring E for which the calculations gave a conformation closer to envelope on C17, instead of the observed twisted conformation around C17–C18.

### Experimental

All reagents were obtained from Sigma-Aldrich Co. THF was dried and purified before use according to standard procedures. A solution of betulin (200 mg, 0.45 mmol) and CDI (219 mg, 1.35 mmol) was refluxed in anyhdrous THF (8 ml). After 7 h the reaction was complete (TLC control). Water (30 ml) was added to the mixture and the resulting precipitate was dissolved in ethyl ether (50 ml). The aqueous phase was extracted twice with diethyl ether (2 *x* 30 ml). The organic phase was then washed with water (30 ml), brine (30 ml), dried with anhydrous Na_2_SO_4_, filtered, and concentrated under reduced pressure to give a yellowish solid. This solid was submitted to f.c.c. with petroleum ether 40–60°C/ethyl acetate (3:2) and afforded the title compound (246 mg, 82%). Full analytical details for this compound (MS, IR, ^1^H and ^13^C NMR spectroscopy data) can be found in Santos *et al.*, 2009. Recrystallization from acetone at room temperature gave colourless single crystals suitable for X-ray diffraction.

*Ab initio* calculations were based on a molecular orbital Roothaan Hartree-Fock method using an extended 6–31 G(d,p) basis set. Tight conditions for convergence of both the self-consistent field cycles and maximum density and energy gradient variations were imposed (10^-6^ atomic units). The program was run on the Milipeia cluster of UC-LCA (using 16 Opteron cores at 2.2 GHz, runing Linux).

### Refinement

All H atoms attached to C atoms were refined as riding on their parent atoms using *SHELXL97* defaults. The H atom of the hydroxyl group was refined using an HFIX 147 instruction with *U*_iso_= 1.5 *U*_eq_ of the O atom. The absolute configuration was not determined from the X-ray data, as the molecule lacks any strong anomalous scatterers at the Mo Kα wavelength, but was known from the synthetic route. Friedel pairs were merged for the refinement.

### Crystal data

**Table:**

C_34_H_52_N_2_O_3_	*D*_x_ = 1.197 Mg m^−^^3^
*M**_r_* = 536.78	Melting point: 476 K
Orthorhombic, *P*2_1_2_1_2_1_	Mo *K*α radiation, λ = 0.71073 Å
Hall symbol: P 2ac 2ab	Cell parameters from 3394 reflections
*a* = 8.2575 (2) Å	θ = 2.6–19.6°
*b* = 12.3909 (4) Å	µ = 0.08 mm^−^^1^
*c* = 29.0992 (8) Å	*T* = 293 K
*V* = 2977.37 (15) Å^3^	Block, colourless
*Z* = 4	0.25 × 0.22 × 0.18 mm
*F*(000) = 1176	

### Data collection

**Table:**

Bruker APEXII CCD area-detector diffractometer	3117 independent reflections
Radiation source: fine-focus sealed tube	2106 reflections with *I* > 2σ(*I*)
graphite	*R*_int_ = 0.111
φ and ω scans	θ_max_ = 25.4°, θ_min_ = 1.4°
Absorption correction: multi-scan (*SADABS*; Sheldrick, 2000)	*h* = −9→9
*T*_min_ = 0.898, *T*_max_ = 1.0	*k* = −14→14
54547 measured reflections	*l* = −35→35

### Refinement

**Table:**

Refinement on *F*^2^	Secondary atom site location: difference Fourier map
Least-squares matrix: full	Hydrogen site location: inferred from neighbouring sites
*R*[*F*^2^ > 2σ(*F*^2^)] = 0.049	H-atom parameters constrained
*wR*(*F*^2^) = 0.119	*w* = 1/[σ^2^(*F*_o_^2^) + (0.0588*P*)^2^ + 0.259*P*] where *P* = (*F*_o_^2^ + 2*F*_c_^2^)/3
*S* = 1.02	(Δ/σ)_max_ < 0.001
3117 reflections	Δρ_max_ = 0.17 e Å^−^^3^
360 parameters	Δρ_min_ = −0.19 e Å^−^^3^
0 restraints	Extinction correction: *SHELXL97* (Sheldrick, 2008), Fc^*^=kFc[1+0.001xFc^2^λ^3^/sin(2θ)]^-1/4^
Primary atom site location: structure-invariant direct methods	Extinction coefficient: 0.0030 (6)

### Special details

**Table:**

Geometry. All e.s.d.'s (except the e.s.d. in the dihedral angle between two l.s. planes) are estimated using the full covariance matrix. The cell e.s.d.'s are taken into account individually in the estimation of e.s.d.'s in distances, angles and torsion angles; correlations between e.s.d.'s in cell parameters are only used when they are defined by crystal symmetry. An approximate (isotropic) treatment of cell e.s.d.'s is used for estimating e.s.d.'s involving l.s. planes.
Refinement. Refinement of *F*^2^ against ALL reflections. The weighted *R*-factor *wR* and goodness of fit *S* are based on *F*^2^, conventional *R*-factors *R* are based on *F*, with *F* set to zero for negative *F*^2^. The threshold expression of *F*^2^ > σ(*F*^2^) is used only for calculating *R*-factors(gt) *etc*. and is not relevant to the choice of reflections for refinement. *R*-factors based on *F*^2^ are statistically about twice as large as those based on *F*, and *R*- factors based on ALL data will be even larger.

### Fractional atomic coordinates and isotropic or equivalent isotropic displacement parameters (Å^2^)

**Table:**

	*x*	*y*	*z*	*U*_iso_*/*U*_eq_	
O3A	1.2324 (3)	0.9802 (3)	−0.21004 (8)	0.0692 (9)	
H3A	1.1856	0.9476	−0.2307	0.104*	
O28A	0.3667 (3)	1.0883 (2)	0.12637 (8)	0.0487 (7)	
O28B	0.3861 (3)	1.1078 (3)	0.20317 (9)	0.0608 (8)	
N28A	0.1558 (3)	1.1559 (2)	0.16449 (11)	0.0456 (8)	
N28B	−0.0758 (4)	1.2165 (3)	0.13709 (15)	0.0720 (11)	
C1	1.1881 (4)	0.9825 (3)	−0.08289 (11)	0.0431 (10)	
H1A	1.2619	1.0103	−0.0598	0.052*	
H1B	1.1784	0.9052	−0.0782	0.052*	
C2	1.2598 (4)	1.0030 (4)	−0.13052 (11)	0.0472 (10)	
H2A	1.2766	1.0799	−0.1346	0.057*	
H2B	1.3643	0.9676	−0.1328	0.057*	
C3	1.1511 (4)	0.9619 (3)	−0.16763 (11)	0.0447 (9)	
H3	1.1410	0.8837	−0.1636	0.054*	
C4	0.9786 (4)	1.0102 (3)	−0.16640 (12)	0.0403 (9)	
C5	0.9112 (4)	0.9973 (3)	−0.11667 (11)	0.0351 (8)	
H5	0.9023	0.9191	−0.1124	0.042*	
C6	0.7385 (4)	1.0385 (3)	−0.11032 (11)	0.0449 (10)	
H6A	0.7396	1.1166	−0.1081	0.054*	
H6B	0.6739	1.0187	−0.1369	0.054*	
C7	0.6626 (4)	0.9911 (3)	−0.06708 (11)	0.0452 (10)	
H7A	0.6511	0.9138	−0.0711	0.054*	
H7B	0.5549	1.0211	−0.0634	0.054*	
C8	0.7600 (4)	1.0123 (3)	−0.02291 (11)	0.0354 (8)	
C9	0.9435 (4)	0.9876 (3)	−0.03140 (11)	0.0342 (8)	
H9	0.9479	0.9092	−0.0358	0.041*	
C10	1.0205 (4)	1.0351 (3)	−0.07616 (11)	0.0350 (8)	
C11	1.0414 (4)	1.0077 (3)	0.01245 (11)	0.0421 (9)	
H11A	1.1537	0.9890	0.0069	0.051*	
H11B	1.0369	1.0839	0.0200	0.051*	
C12	0.9792 (4)	0.9427 (3)	0.05311 (11)	0.0390 (9)	
H12A	1.0394	0.9628	0.0804	0.047*	
H12B	0.9980	0.8666	0.0474	0.047*	
C13	0.7987 (4)	0.9610 (3)	0.06169 (10)	0.0334 (8)	
H13	0.7858	1.0383	0.0678	0.040*	
C14	0.6979 (4)	0.9367 (3)	0.01754 (11)	0.0346 (8)	
C15	0.5141 (4)	0.9542 (3)	0.02663 (12)	0.0455 (10)	
H15A	0.4933	1.0311	0.0280	0.055*	
H15B	0.4539	0.9255	0.0007	0.055*	
C16	0.4497 (4)	0.9020 (3)	0.07071 (12)	0.0470 (10)	
H16A	0.3380	0.9237	0.0754	0.056*	
H16B	0.4520	0.8241	0.0674	0.056*	
C17	0.5496 (4)	0.9344 (3)	0.11233 (12)	0.0376 (9)	
C18	0.7289 (4)	0.9020 (3)	0.10367 (11)	0.0365 (9)	
H18	0.7282	0.8250	0.0959	0.044*	
C19	0.8096 (4)	0.9114 (3)	0.15107 (11)	0.0403 (9)	
H19	0.8379	0.9874	0.1560	0.048*	
C20	0.9578 (5)	0.8441 (3)	0.16120 (13)	0.0477 (10)	
C21	0.6699 (5)	0.8817 (4)	0.18491 (13)	0.0552 (11)	
H21A	0.6939	0.8144	0.2005	0.066*	
H21B	0.6571	0.9379	0.2079	0.066*	
C22	0.5159 (4)	0.8707 (3)	0.15669 (13)	0.0466 (10)	
H22A	0.4240	0.9008	0.1730	0.056*	
H22B	0.4938	0.7955	0.1499	0.056*	
C23	0.9800 (6)	1.1274 (3)	−0.18353 (14)	0.0609 (12)	
H23A	1.0188	1.1295	−0.2146	0.091*	
H23B	1.0499	1.1699	−0.1643	0.091*	
H23C	0.8722	1.1563	−0.1824	0.091*	
C24	0.8719 (5)	0.9437 (4)	−0.19920 (12)	0.0549 (11)	
H24A	0.7664	0.9760	−0.2012	0.082*	
H24B	0.8620	0.8714	−0.1877	0.082*	
H24C	0.9206	0.9422	−0.2291	0.082*	
C25	1.0456 (5)	1.1586 (3)	−0.07340 (13)	0.0534 (11)	
H25A	1.0781	1.1780	−0.0428	0.080*	
H25B	0.9461	1.1946	−0.0809	0.080*	
H25C	1.1282	1.1799	−0.0948	0.080*	
C26	0.7317 (5)	1.1319 (3)	−0.01023 (12)	0.0494 (10)	
H26A	0.7605	1.1768	−0.0358	0.074*	
H26B	0.7974	1.1505	0.0158	0.074*	
H26C	0.6196	1.1427	−0.0028	0.074*	
C27	0.7156 (5)	0.8154 (3)	0.00488 (12)	0.0455 (10)	
H27A	0.6582	0.7723	0.0269	0.068*	
H27B	0.8281	0.7959	0.0051	0.068*	
H27C	0.6715	0.8031	−0.0252	0.068*	
C28	0.5360 (4)	1.0553 (3)	0.12224 (13)	0.0461 (10)	
H28A	0.5927	1.0719	0.1506	0.055*	
H28B	0.5870	1.0956	0.0976	0.055*	
C28A	0.3144 (4)	1.1153 (3)	0.16739 (14)	0.0452 (9)	
C28B	0.0639 (5)	1.1753 (3)	0.12673 (15)	0.0536 (11)	
H28C	0.0974	1.1608	0.0968	0.064*	
C28C	−0.0750 (6)	1.2231 (4)	0.18384 (19)	0.0810 (16)	
H28D	−0.1615	1.2487	0.2012	0.097*	
C28D	0.0650 (5)	1.1884 (4)	0.20191 (16)	0.0702 (14)	
H28E	0.0945	1.1867	0.2327	0.084*	
C29	0.9945 (6)	0.7521 (4)	0.13855 (17)	0.0801 (15)	
H29A	1.0819	0.7103	0.1480	0.096*	
H29B	0.9324	0.7306	0.1135	0.096*	
C30	1.0532 (6)	0.8776 (4)	0.19981 (16)	0.0818 (16)	
H30A	1.1456	0.8311	0.2028	0.123*	
H30B	0.9890	0.8735	0.2273	0.123*	
H30C	1.0889	0.9506	0.1953	0.123*	

### Atomic displacement parameters (Å^2^)

**Table:**

	*U*^11^	*U*^22^	*U*^33^	*U*^12^	*U*^13^	*U*^23^
O3A	0.0532 (18)	0.118 (3)	0.0366 (15)	−0.0123 (18)	0.0124 (13)	−0.0020 (16)
O28A	0.0432 (15)	0.0619 (18)	0.0411 (15)	0.0164 (13)	0.0093 (12)	−0.0033 (14)
O28B	0.0542 (17)	0.083 (2)	0.0449 (17)	0.0161 (16)	−0.0023 (14)	0.0079 (16)
N28A	0.0382 (16)	0.0482 (19)	0.0505 (19)	0.0132 (15)	0.0066 (16)	0.0009 (17)
N28B	0.054 (2)	0.071 (3)	0.091 (3)	0.020 (2)	−0.016 (2)	−0.009 (2)
C1	0.0292 (18)	0.063 (3)	0.038 (2)	−0.0043 (19)	−0.0011 (15)	0.0032 (19)
C2	0.034 (2)	0.070 (3)	0.038 (2)	−0.005 (2)	0.0047 (16)	0.001 (2)
C3	0.041 (2)	0.060 (3)	0.034 (2)	−0.0035 (18)	0.0061 (18)	0.0000 (19)
C4	0.043 (2)	0.048 (2)	0.0300 (19)	−0.0036 (18)	−0.0006 (17)	0.0017 (18)
C5	0.0328 (18)	0.040 (2)	0.0322 (19)	−0.0014 (17)	−0.0038 (15)	0.0012 (17)
C6	0.038 (2)	0.058 (3)	0.039 (2)	0.0024 (19)	−0.0030 (17)	0.0054 (19)
C7	0.0283 (17)	0.065 (3)	0.042 (2)	0.0008 (19)	−0.0018 (16)	−0.001 (2)
C8	0.0311 (18)	0.039 (2)	0.0362 (19)	0.0052 (16)	−0.0016 (15)	−0.0024 (17)
C9	0.0282 (17)	0.043 (2)	0.0319 (19)	−0.0013 (16)	−0.0053 (15)	−0.0007 (16)
C10	0.0297 (19)	0.040 (2)	0.035 (2)	−0.0021 (16)	0.0007 (16)	−0.0016 (16)
C11	0.0297 (18)	0.065 (3)	0.0313 (19)	−0.0065 (19)	−0.0005 (15)	−0.0022 (19)
C12	0.0309 (18)	0.052 (2)	0.034 (2)	0.0016 (18)	−0.0033 (16)	−0.0058 (17)
C13	0.0308 (18)	0.038 (2)	0.0311 (18)	−0.0021 (15)	0.0020 (15)	−0.0038 (16)
C14	0.0269 (17)	0.042 (2)	0.0349 (19)	−0.0031 (16)	0.0042 (15)	−0.0044 (16)
C15	0.0309 (19)	0.062 (3)	0.043 (2)	0.0027 (18)	−0.0013 (17)	−0.0008 (19)
C16	0.0302 (18)	0.060 (3)	0.050 (2)	0.0003 (19)	0.0032 (18)	−0.002 (2)
C17	0.036 (2)	0.036 (2)	0.041 (2)	0.0021 (17)	0.0088 (17)	−0.0011 (17)
C18	0.0317 (18)	0.040 (2)	0.038 (2)	0.0027 (16)	0.0008 (16)	−0.0015 (17)
C19	0.042 (2)	0.040 (2)	0.039 (2)	−0.0010 (17)	−0.0004 (17)	−0.0028 (18)
C20	0.044 (2)	0.056 (3)	0.043 (2)	−0.002 (2)	0.004 (2)	0.016 (2)
C21	0.060 (3)	0.062 (3)	0.045 (2)	0.002 (2)	0.007 (2)	0.005 (2)
C22	0.043 (2)	0.045 (2)	0.051 (2)	0.0038 (18)	0.0128 (19)	0.0057 (19)
C23	0.070 (3)	0.060 (3)	0.053 (3)	0.003 (2)	−0.002 (2)	0.021 (2)
C24	0.049 (2)	0.076 (3)	0.040 (2)	−0.008 (2)	−0.0032 (19)	0.000 (2)
C25	0.063 (3)	0.048 (3)	0.049 (2)	−0.015 (2)	0.003 (2)	−0.006 (2)
C26	0.050 (2)	0.049 (3)	0.050 (2)	0.006 (2)	0.0109 (19)	0.0034 (19)
C27	0.045 (2)	0.045 (2)	0.046 (2)	−0.0104 (19)	−0.0012 (19)	−0.0084 (18)
C28	0.038 (2)	0.052 (3)	0.047 (2)	0.0067 (19)	0.0117 (18)	0.0009 (19)
C28A	0.042 (2)	0.044 (2)	0.050 (3)	0.0037 (19)	0.005 (2)	0.005 (2)
C28B	0.057 (3)	0.050 (3)	0.054 (3)	0.002 (2)	−0.010 (2)	−0.004 (2)
C28C	0.057 (3)	0.098 (4)	0.088 (4)	0.033 (3)	0.009 (3)	−0.011 (3)
C28D	0.061 (3)	0.095 (4)	0.054 (3)	0.024 (3)	0.008 (2)	−0.009 (3)
C29	0.076 (3)	0.069 (3)	0.095 (4)	0.028 (3)	−0.025 (3)	−0.018 (3)
C30	0.064 (3)	0.110 (4)	0.072 (3)	0.004 (3)	−0.010 (3)	−0.004 (3)

### Geometric parameters (Å, °)

**Table:**

O3A—C3	1.423 (4)	C14—C15	1.555 (4)
O3A—H3A	0.8200	C15—C16	1.532 (5)
O28A—C28A	1.312 (4)	C15—H15A	0.9700
O28A—C28	1.462 (4)	C15—H15B	0.9700
O28B—C28A	1.201 (4)	C16—C17	1.520 (5)
N28A—C28B	1.357 (5)	C16—H16A	0.9700
N28A—C28D	1.382 (5)	C16—H16B	0.9700
N28A—C28A	1.406 (5)	C17—C28	1.529 (5)
N28B—C28B	1.297 (5)	C17—C22	1.539 (5)
N28B—C28C	1.363 (6)	C17—C18	1.555 (5)
C1—C2	1.528 (4)	C18—C19	1.536 (4)
C1—C10	1.543 (5)	C18—H18	0.9800
C1—H1A	0.9700	C19—C20	1.510 (5)
C1—H1B	0.9700	C19—C21	1.561 (5)
C2—C3	1.494 (5)	C19—H19	0.9800
C2—H2A	0.9700	C20—C29	1.352 (6)
C2—H2B	0.9700	C20—C30	1.433 (6)
C3—C4	1.546 (5)	C21—C22	1.520 (5)
C3—H3	0.9800	C21—H21A	0.9700
C4—C23	1.535 (5)	C21—H21B	0.9700
C4—C24	1.539 (5)	C22—H22A	0.9700
C4—C5	1.559 (5)	C22—H22B	0.9700
C5—C6	1.526 (5)	C23—H23A	0.9600
C5—C10	1.557 (5)	C23—H23B	0.9600
C5—H5	0.9800	C23—H23C	0.9600
C6—C7	1.524 (4)	C24—H24A	0.9600
C6—H6A	0.9700	C24—H24B	0.9600
C6—H6B	0.9700	C24—H24C	0.9600
C7—C8	1.539 (5)	C25—H25A	0.9600
C7—H7A	0.9700	C25—H25B	0.9600
C7—H7B	0.9700	C25—H25C	0.9600
C8—C26	1.545 (5)	C26—H26A	0.9600
C8—C9	1.566 (4)	C26—H26B	0.9600
C8—C14	1.590 (5)	C26—H26C	0.9600
C9—C11	1.531 (4)	C27—H27A	0.9600
C9—C10	1.565 (4)	C27—H27B	0.9600
C9—H9	0.9800	C27—H27C	0.9600
C10—C25	1.547 (5)	C28—H28A	0.9700
C11—C12	1.520 (4)	C28—H28B	0.9700
C11—H11A	0.9700	C28B—H28C	0.9300
C11—H11B	0.9700	C28C—C28D	1.341 (6)
C12—C13	1.528 (4)	C28C—H28D	0.9300
C12—H12A	0.9700	C28D—H28E	0.9300
C12—H12B	0.9700	C29—H29A	0.9300
C13—C18	1.536 (4)	C29—H29B	0.9300
C13—C14	1.560 (4)	C30—H30A	0.9600
C13—H13	0.9800	C30—H30B	0.9600
C14—C27	1.554 (5)	C30—H30C	0.9600
			
C3—O3A—H3A	109.5	C17—C16—C15	111.5 (3)
C28A—O28A—C28	117.4 (3)	C17—C16—H16A	109.3
C28B—N28A—C28D	106.5 (3)	C15—C16—H16A	109.3
C28B—N28A—C28A	129.3 (4)	C17—C16—H16B	109.3
C28D—N28A—C28A	124.2 (4)	C15—C16—H16B	109.3
C28B—N28B—C28C	104.6 (4)	H16A—C16—H16B	108.0
C2—C1—C10	113.1 (3)	C16—C17—C28	111.7 (3)
C2—C1—H1A	109.0	C16—C17—C22	115.8 (3)
C10—C1—H1A	109.0	C28—C17—C22	109.3 (3)
C2—C1—H1B	109.0	C16—C17—C18	108.6 (3)
C10—C1—H1B	109.0	C28—C17—C18	110.7 (3)
H1A—C1—H1B	107.8	C22—C17—C18	100.1 (3)
C3—C2—C1	111.5 (3)	C13—C18—C19	121.0 (3)
C3—C2—H2A	109.3	C13—C18—C17	111.3 (3)
C1—C2—H2A	109.3	C19—C18—C17	104.4 (3)
C3—C2—H2B	109.3	C13—C18—H18	106.4
C1—C2—H2B	109.3	C19—C18—H18	106.4
H2A—C2—H2B	108.0	C17—C18—H18	106.4
O3A—C3—C2	106.8 (3)	C20—C19—C18	119.0 (3)
O3A—C3—C4	113.1 (3)	C20—C19—C21	110.2 (3)
C2—C3—C4	113.9 (3)	C18—C19—C21	103.2 (3)
O3A—C3—H3	107.6	C20—C19—H19	108.0
C2—C3—H3	107.6	C18—C19—H19	108.0
C4—C3—H3	107.6	C21—C19—H19	108.0
C23—C4—C24	108.0 (3)	C29—C20—C30	120.2 (4)
C23—C4—C3	110.6 (3)	C29—C20—C19	123.5 (4)
C24—C4—C3	107.8 (3)	C30—C20—C19	116.0 (4)
C23—C4—C5	113.7 (3)	C22—C21—C19	107.4 (3)
C24—C4—C5	108.4 (3)	C22—C21—H21A	110.2
C3—C4—C5	108.1 (3)	C19—C21—H21A	110.2
C6—C5—C10	110.4 (3)	C22—C21—H21B	110.2
C6—C5—C4	114.3 (3)	C19—C21—H21B	110.2
C10—C5—C4	117.7 (3)	H21A—C21—H21B	108.5
C6—C5—H5	104.2	C21—C22—C17	104.8 (3)
C10—C5—H5	104.2	C21—C22—H22A	110.8
C4—C5—H5	104.2	C17—C22—H22A	110.8
C7—C6—C5	110.8 (3)	C21—C22—H22B	110.8
C7—C6—H6A	109.5	C17—C22—H22B	110.8
C5—C6—H6A	109.5	H22A—C22—H22B	108.9
C7—C6—H6B	109.5	C4—C23—H23A	109.5
C5—C6—H6B	109.5	C4—C23—H23B	109.5
H6A—C6—H6B	108.1	H23A—C23—H23B	109.5
C6—C7—C8	114.1 (3)	C4—C23—H23C	109.5
C6—C7—H7A	108.7	H23A—C23—H23C	109.5
C8—C7—H7A	108.7	H23B—C23—H23C	109.5
C6—C7—H7B	108.7	C4—C24—H24A	109.5
C8—C7—H7B	108.7	C4—C24—H24B	109.5
H7A—C7—H7B	107.6	H24A—C24—H24B	109.5
C7—C8—C26	106.5 (3)	C4—C24—H24C	109.5
C7—C8—C9	109.9 (3)	H24A—C24—H24C	109.5
C26—C8—C9	111.8 (3)	H24B—C24—H24C	109.5
C7—C8—C14	110.4 (3)	C10—C25—H25A	109.5
C26—C8—C14	109.9 (3)	C10—C25—H25B	109.5
C9—C8—C14	108.3 (3)	H25A—C25—H25B	109.5
C11—C9—C10	114.7 (3)	C10—C25—H25C	109.5
C11—C9—C8	110.3 (3)	H25A—C25—H25C	109.5
C10—C9—C8	116.8 (3)	H25B—C25—H25C	109.5
C11—C9—H9	104.5	C8—C26—H26A	109.5
C10—C9—H9	104.5	C8—C26—H26B	109.5
C8—C9—H9	104.5	H26A—C26—H26B	109.5
C1—C10—C25	107.8 (3)	C8—C26—H26C	109.5
C1—C10—C5	107.2 (3)	H26A—C26—H26C	109.5
C25—C10—C5	114.5 (3)	H26B—C26—H26C	109.5
C1—C10—C9	108.1 (3)	C14—C27—H27A	109.5
C25—C10—C9	112.6 (3)	C14—C27—H27B	109.5
C5—C10—C9	106.3 (3)	H27A—C27—H27B	109.5
C12—C11—C9	112.6 (3)	C14—C27—H27C	109.5
C12—C11—H11A	109.1	H27A—C27—H27C	109.5
C9—C11—H11A	109.1	H27B—C27—H27C	109.5
C12—C11—H11B	109.1	O28A—C28—C17	111.1 (3)
C9—C11—H11B	109.1	O28A—C28—H28A	109.4
H11A—C11—H11B	107.8	C17—C28—H28A	109.4
C11—C12—C13	112.2 (3)	O28A—C28—H28B	109.4
C11—C12—H12A	109.2	C17—C28—H28B	109.4
C13—C12—H12A	109.2	H28A—C28—H28B	108.0
C11—C12—H12B	109.2	O28B—C28A—O28A	127.3 (3)
C13—C12—H12B	109.2	O28B—C28A—N28A	122.6 (4)
H12A—C12—H12B	107.9	O28A—C28A—N28A	110.1 (3)
C12—C13—C18	115.1 (3)	N28B—C28B—N28A	112.2 (4)
C12—C13—C14	110.9 (3)	N28B—C28B—H28C	123.9
C18—C13—C14	111.3 (3)	N28A—C28B—H28C	123.9
C12—C13—H13	106.3	C28D—C28C—N28B	112.1 (4)
C18—C13—H13	106.3	C28D—C28C—H28D	124.0
C14—C13—H13	106.3	N28B—C28C—H28D	124.0
C27—C14—C15	105.5 (3)	C28C—C28D—N28A	104.6 (4)
C27—C14—C13	109.4 (3)	C28C—C28D—H28E	127.7
C15—C14—C13	110.7 (3)	N28A—C28D—H28E	127.7
C27—C14—C8	111.4 (3)	C20—C29—H29A	120.0
C15—C14—C8	111.0 (3)	C20—C29—H29B	120.0
C13—C14—C8	108.9 (3)	H29A—C29—H29B	120.0
C16—C15—C14	115.0 (3)	C20—C30—H30A	109.5
C16—C15—H15A	108.5	C20—C30—H30B	109.5
C14—C15—H15A	108.5	H30A—C30—H30B	109.5
C16—C15—H15B	108.5	C20—C30—H30C	109.5
C14—C15—H15B	108.5	H30A—C30—H30C	109.5
H15A—C15—H15B	107.5	H30B—C30—H30C	109.5
			
C10—C1—C2—C3	−58.5 (4)	C9—C8—C14—C27	−60.7 (3)
C1—C2—C3—O3A	−177.2 (3)	C7—C8—C14—C15	−57.6 (4)
C1—C2—C3—C4	57.1 (4)	C26—C8—C14—C15	59.6 (4)
O3A—C3—C4—C23	−48.1 (4)	C9—C8—C14—C15	−178.0 (3)
C2—C3—C4—C23	74.1 (4)	C7—C8—C14—C13	−179.7 (3)
O3A—C3—C4—C24	69.8 (4)	C26—C8—C14—C13	−62.5 (3)
C2—C3—C4—C24	−168.0 (3)	C9—C8—C14—C13	59.9 (3)
O3A—C3—C4—C5	−173.2 (3)	C27—C14—C15—C16	70.6 (4)
C2—C3—C4—C5	−51.0 (4)	C13—C14—C15—C16	−47.6 (4)
C23—C4—C5—C6	58.6 (4)	C8—C14—C15—C16	−168.6 (3)
C24—C4—C5—C6	−61.6 (4)	C14—C15—C16—C17	52.6 (4)
C3—C4—C5—C6	−178.2 (3)	C15—C16—C17—C28	65.0 (4)
C23—C4—C5—C10	−73.5 (4)	C15—C16—C17—C22	−169.0 (3)
C24—C4—C5—C10	166.3 (3)	C15—C16—C17—C18	−57.4 (4)
C3—C4—C5—C10	49.7 (4)	C12—C13—C18—C19	51.4 (4)
C10—C5—C6—C7	−63.2 (4)	C14—C13—C18—C19	178.7 (3)
C4—C5—C6—C7	161.3 (3)	C12—C13—C18—C17	174.4 (3)
C5—C6—C7—C8	56.1 (4)	C14—C13—C18—C17	−58.3 (4)
C6—C7—C8—C26	75.0 (4)	C16—C17—C18—C13	61.6 (4)
C6—C7—C8—C9	−46.3 (4)	C28—C17—C18—C13	−61.4 (4)
C6—C7—C8—C14	−165.7 (3)	C22—C17—C18—C13	−176.7 (3)
C7—C8—C9—C11	−179.6 (3)	C16—C17—C18—C19	−166.3 (3)
C26—C8—C9—C11	62.3 (4)	C28—C17—C18—C19	70.7 (4)
C14—C8—C9—C11	−58.9 (4)	C22—C17—C18—C19	−44.6 (3)
C7—C8—C9—C10	47.0 (4)	C13—C18—C19—C20	−79.6 (4)
C26—C8—C9—C10	−71.1 (4)	C17—C18—C19—C20	154.2 (3)
C14—C8—C9—C10	167.7 (3)	C13—C18—C19—C21	158.0 (3)
C2—C1—C10—C25	−70.7 (4)	C17—C18—C19—C21	31.8 (4)
C2—C1—C10—C5	53.0 (4)	C18—C19—C20—C29	−22.7 (5)
C2—C1—C10—C9	167.4 (3)	C21—C19—C20—C29	96.1 (5)
C6—C5—C10—C1	175.3 (3)	C18—C19—C20—C30	163.0 (4)
C4—C5—C10—C1	−51.0 (4)	C21—C19—C20—C30	−78.2 (4)
C6—C5—C10—C25	−65.2 (4)	C20—C19—C21—C22	−134.8 (3)
C4—C5—C10—C25	68.5 (4)	C18—C19—C21—C22	−6.7 (4)
C6—C5—C10—C9	59.8 (4)	C19—C21—C22—C17	−21.2 (4)
C4—C5—C10—C9	−166.5 (3)	C16—C17—C22—C21	156.3 (3)
C11—C9—C10—C1	60.2 (4)	C28—C17—C22—C21	−76.5 (4)
C8—C9—C10—C1	−168.4 (3)	C18—C17—C22—C21	39.8 (4)
C11—C9—C10—C25	−58.7 (4)	C28A—O28A—C28—C17	109.7 (4)
C8—C9—C10—C25	72.7 (4)	C16—C17—C28—O28A	53.7 (4)
C11—C9—C10—C5	175.1 (3)	C22—C17—C28—O28A	−75.7 (4)
C8—C9—C10—C5	−53.5 (4)	C18—C17—C28—O28A	174.9 (3)
C10—C9—C11—C12	−169.2 (3)	C28—O28A—C28A—O28B	−6.5 (6)
C8—C9—C11—C12	56.4 (4)	C28—O28A—C28A—N28A	173.6 (3)
C9—C11—C12—C13	−54.2 (4)	C28B—N28A—C28A—O28B	175.4 (4)
C11—C12—C13—C18	−177.3 (3)	C28D—N28A—C28A—O28B	−1.8 (6)
C11—C12—C13—C14	55.2 (4)	C28B—N28A—C28A—O28A	−4.7 (5)
C12—C13—C14—C27	63.5 (4)	C28D—N28A—C28A—O28A	178.1 (4)
C18—C13—C14—C27	−66.0 (3)	C28C—N28B—C28B—N28A	−0.6 (5)
C12—C13—C14—C15	179.4 (3)	C28D—N28A—C28B—N28B	−0.3 (5)
C18—C13—C14—C15	49.8 (4)	C28A—N28A—C28B—N28B	−177.9 (4)
C12—C13—C14—C8	−58.4 (4)	C28B—N28B—C28C—C28D	1.3 (7)
C18—C13—C14—C8	172.1 (3)	N28B—C28C—C28D—N28A	−1.4 (6)
C7—C8—C14—C27	59.6 (4)	C28B—N28A—C28D—C28C	1.0 (5)
C26—C8—C14—C27	176.9 (3)	C28A—N28A—C28D—C28C	178.8 (4)

### Hydrogen-bond geometry (Å, °)

**Table:**

*D*—H···*A*	*D*—H	H···*A*	*D*···*A*	*D*—H···*A*
O3A—H3A···O28B^i^	0.82	2.13	2.920 (4)	162

Symmetry codes: (i) −*x*+3/2, −*y*+2, *z*−1/2.
